# Effectiveness of Body Psychotherapy. A Systematic Review and Meta-Analysis

**DOI:** 10.3389/fpsyt.2021.709798

**Published:** 2021-09-09

**Authors:** Sophie Rosendahl, Heribert Sattel, Claas Lahmann

**Affiliations:** ^1^Department of Pneumology and Respiratory Medicine, Thoraxklinik, University of Heidelberg, Heidelberg, Germany; ^2^Department of Psychosomatic Medicine and Psychotherapy, Klinikum Rechts der Isar, Technical University Munich, Munich, Germany; ^3^Faculty of Medicine, Department of Psychosomatic Medicine and Psychotherapy, Medical Center—University of Freiburg, University of Freiburg, Freiburg im Breisgau, Germany

**Keywords:** body psychotherapy, embodiment, systematic review, meta-analysis, psychic distress

## Abstract

Despite the growing relevance and applicability of elements based on and derived from the *embodied mind* paradigm, body psychotherapy (BPT) appears not to be a well-established treatment option. This might be due to a lack of proof for its efficacy. We searched electronic databases (Pubmed MEDLINE, PsycInfo, and PSYNDEX) for randomized controlled trials (RCTs) examining predefined BPT interventions. A total of 2,180 references were screened, of which 113 studies were scrutinized in detail and 18 RCTs finally included. The observed effect size (ES) demonstrated medium effects of BPT on primary outcomes psychopathology and psychological distress. In case of significant statistical heterogeneity, exploratory subgroup analyses revealed diagnosis and the degree of control group activity as noteworthy moderators. For secondary outcomes, evidence was scarce, and an improvement could be demonstrated only for coping abilities. The identified evidence indicates that BPT is beneficial for a wide spectrum of psychic suffering. There is a strong need for high-quality studies with bigger samples and for well-defined diagnostic entities to underpin its effectiveness.

## Introduction

Feeling and connecting to one's own body plays a central role in psychotherapy since its beginning: the founders of psychoanalytic therapy regarded somatic tensions as an expression of mental conflicts (1, 2), and in retrospect, the gymnastic movement in the early nineteenth century was the cornerstone for movement- and body-centered techniques (3). Nowadays, the popularity of trainings claiming the propagation of mindfulness such as Yoga, Pilates, Qigong, and progressive muscle relaxation draws its persuasiveness out of the common intuition that a phenomenal integration of body and mind is indispensable for a person's well-being. On the one hand, the body constitutes the stage where mental disorders develop and unfold: in primary care, the majority of patients with psychiatric disorders presents somatic symptoms (4–6). On the other hand, it is the precondition of the psychotherapeutic process in which patient and therapist communicate not only verbally but also in a body dialogue (3). Consequently, in a survey, 88% of German psychosomatic clinics stated to employ body-oriented therapies (7), and the majority of chief physicians stresses its importance in the field (8). Despite this omnipresence in practice, psychotherapeutic techniques that focus on the body are neglected in the scientific discourse (9, 10). There is a lack of summarizing proof on the specific contribution of psychotherapy employing body techniques following the standards of evidence-based medicine.

### Body Experience in Psychotherapy

In psychotherapy, an ambiguity toward bodily experiences becomes apparent that stems from the phenomenological consideration of the body as subject as opposed to the body as object: whereas the former means the experience of the body as constituting the first-person perspective from which an individual encounters the world, the latter refers to the objectified body that natural science examines on different levels (biochemical, neuronal, molecular, etc.) (11–13). Although this epistemological distinction might be neglected in psychotherapy, its consideration may lead to a gain in explanatory power from the possible phenomenological change between both perspectives involved. Likewise, Gallagher (14) differentiates body schema and body image as opposed stances of an individual. While the former describes the implicit, pre-reflexive role of the body as an inevitable precondition for action, the latter summarizes perceptions and evaluations of the body from an intentional point of view. For others, bodily experiences constitute a spectrum from body-related perceptions and emotions to the evaluation of the own body (15).

In case of pathology, Weizsäcker's model of the “Gestaltkreis” (16) offers an explanation of the invisibility of the psychological conflict of an individual as somatic symptoms replace it. Thus, it is the task of psychotherapy to clarify this shift by means of the complementary perspectives of client and therapist on the client's body. Whereas, the client has a private sphere no one else can enter, the therapist contributes his impression of the client's body, hence an outer perception, interpretation, and own reaction to it. The implied discrepancy between the perspectives is the precondition for the revelation of the unconscious in the intersubjective process (17, 18). In this sense, psychotherapy strives to find a functional explanation and solution in which thought, feeling, body experience, and expression can be intertwined (19). For example, “functional relaxation” understands breathing exercises as a semiotic process, in which the therapist contains the bodily signals of a client, interprets them as his own, and verbalizes the result (20, 21).

### Definition of Body Psychotherapy

Due to its application in diverse and theoretically opposed psychotherapeutic streams, a common definition of BPT is not easy to state. Geuter (3) proposes to define BPT as a treatment employing psychological and bodily means equally. For him, the specificity of BPT lies in the continuous combination of both channels and the interaction of client and therapist on the body level. Loew et al. (22) differentiate *body therapy* and BPT. The former technique focuses on the experienced body to increase the person's physical and psychological well-being. BPT fulfills, in addition, the standards of a verbal psychotherapy following Strotzka (23), which demand a predefined and planned interactional process based on a theoretical foundation, which has clearly defined aims in the treatment of conditions of significant mental suffering or mental diagnoses. Furthermore, it implies verbal and non-verbal psychological means that can be taught and requires a stable emotional relationship between client and therapist. Likewise, for Wampold (24), psychotherapy is foremost an interpersonal treatment. For the purpose of this systematic review and meta-analysis, we hold on the definition of Loew et al. (22) which emphasizes the intersubjectivity of the body experience as the constituting element of BPT.

As mechanisms of change, Lahmann and Weise (25) presume a reduction of arousal and an amelioration of interoception for bodily signals resulting in an enhanced self-awareness and self-efficacy. In order to achieve a better understanding of the bio-psychosocial model, they propose to employ bodily techniques especially in patients presenting with somatic symptoms. Likewise, Röhricht (10) suggests a wide spectrum of application—from affective disorders, eating disorders, somatoform disorders to schizophrenia, i.e., he states that BPT “uniquely modulate[s] emotional processing, affect regulation, movement behavior, and bodily self-awareness.”

### Embodied Mind as a Common Framework

From a theoretical point of view, BPT fits well into the *embodied mind* paradigm (26) that nowadays prevails in cognitive science. According to this stance, human cognition cannot be explained only as a function of a brain but as an interplay of a whole body and its environment (14, 27). Shapiro (28) stresses that information processing begins in the sensorimotor periphery. From this inevitable evolutionary perspective, human experience and behavior should be understood by their genesis and embedment. Likewise, Petzold and Sieper (29) propose a hermeneutic point of view on BPT in which the informed body contains the mental history and state of a person. A historical consideration offers enriching ideas of implied mechanisms of change. In addition to the associative work psychoanalysis contributed to BPT, bioenergetics work with energetic cycles to enhance and finally reduce tension (2, 30) when solving a conflict. More recent approaches extend the technique of transference and countertransference on the body level of communication (31, 32). With regard to the other root of BPT, the gymnastic movement of the twentieth century stressed the healing power of focusing on detailed experience and rhythms of the body and repetition. Nowadays, an integrative body–mind–spirit model assumes, too, the interconnectedness of body and mind and include spirituality as an existential human domain (33). Such connections can be assessed and observed for, e.g., “grounding,” a mechanism commonly agreed in bodily psychotherapy and dance and movement therapy (34). It remains unclear whether such approaches address psychotherapy rather than aiming at multicomponent lifestyle modification programs (35). Röhricht (36) and Geuter (3) propose different grids to structure and integrate therapeutic streams under the umbrella of BPT. Along with this claim, arguments were brought up (37, 38) to introduce a summarizing work factor “body experience” in psychotherapy along with the five factors identified by Grawe (39).

### Existing Evidence

There are few systematic reviews on BPT that mainly focus on distinct therapies such as mindfulness-based stress-reduction therapy (40, 41) and dance therapy (42). This seems to be unsatisfying as those—more active—treatment approaches assembled under the umbrella term BPT are preferable for a broad range of functional complaints including bodily symptoms and psychic suffering (43). Among these few reviews, Grossman et al. (40) draw an ambivalent picture of BPT. May (44), who included also studies with non-clinical samples and “gray” literature, reports positive results in half of effectiveness and efficacy studies and also stresses negative results. Loew et al. (22) report positive effects on symptoms, body perception, and social behavior in eight studies that employed integrated different methods of BPT. For functional relaxation, especially in the treatment of asthma, they found eight controlled, partly randomized studies that state therapeutic success in self-evaluation and physiological parameters. For concentrative movement therapy, Seidler (45) remarks that there are only a few studies; three quasi-experimental studies imply that this method increases well-being and self-consciousness. Röhricht (10) states an increase in evidence and describes a disorder specific benefit of BPT, namely, on schizophrenia. However, he criticizes retrospective studies, small samples, and a lack of RCTs. The, to our knowledge, most recent review, which captures a wide range of body therapies, states positive results for studies offering BPT (46).

### Aim of this Systematic Review and Meta-Analysis

To the best of our knowledge, none of the above-mentioned reviews conducted a meta-analysis on RCTs testing BPT. Accordingly, we identified and included all interventions that integrated body techniques into psychotherapy in the way outlined above. We addressed a broad range of health conditions, defined as “significant psychic suffering,” in order to determine the efficacy of BPT for a wide population of patients.

## Methods

### Inclusion Criteria

In this systematic review and meta-analysis, we included intervention trials that (1) addressed intersubjective body experience in the above-described manner, enframed in psychotherapeutic measures; (2) had a two-factorial design (two groups, measures pre- and post-treatment) with randomized group allocation; (3) publications had to indicate descriptive statistics such as mean and standard deviation or frequencies suitable for the determination of effect sizes; (4) had to be written in English or German and their abstract had to be available via scientific data bases; and (5) the included participants had to be adult (18+ years) and required a diagnosed psychiatric disorder or significant psychic suffering proven by reliable measures (e.g., cutoff values of validated psychometric scales, interviews or a thorough clinical evaluation). There were no restrictions regarding setting (outpatient or inpatient), format (individual or group), dose, and quality of activity applied in the control group.

### Search Methods

Relevant articles were identified by searching PubMed (MEDLINE), PsycInfo, and PSYNDEX on November 4, 2018. We cross-referenced [(body OR bodily) psychotherap^*^] with (clinical trial OR random^*^ OR empirical research OR evaluation studies). In addition, reference lists of the identified review articles and of the European Body Psychotherapy Association were scrutinized to include further studies. An updated hand search until April 30, 2021 did not detect recent relevant studies. Study selection was carried out by SR and HS, applying a short checklist for the abovementioned criteria. Doubtful cases were discussed between the authors and discrepancies solved by Delphi method, if necessary, under consultation of CL.

As an indicator of the studies' quality, we used the Cochrane Collaboration Depression, Anxiety, and Neurosis (CCDAN) quality assessment rating scale (47), which allows a detailed description of the intervention, of inclusion criteria, and of the study execution, resulting in a quantitative measure of the study quality (Supplementary Table 1). Independent ratings were simultaneously carried out by SR and HS.

### Data Collection and Analysis

To evaluate the effectiveness of the intervention, we calculated effect sizes [standardized mean differences (SMD), Hedges' g] based on the random-effects model between groups on the primary and secondary outcomes. In this way, we accounted for the plausible variability of effects among studies. In case an outcome of interest was reported by more than one scale within a study, we integrated those using z-transformation to avoid multiple testing (48). Per convention, the effect size was interpreted as small if 0.2 ≤ |ES| < 0.5, medium effect if 0.5 ≥ |ES| < 0.8, and large effect if |ES| ≥ 0.8 (49). An effect size higher than 0.5 was judged as clinically relevant (50). We estimated within group changes as well to analyze the absolute effect of an intervention and to exclude that between-group effects were not based on a deterioration of the symptomatology of the control group.

As primary outcomes, we chose measures of psychopathology and psychological distress. Whereas, the former depicts the main characteristic(s) of the psychic suffering, the latter describes the overall functional impairment due to mental or bodily symptoms. As secondary outcomes, measures of coping abilities, quality of life, body experience, and interpersonal difficulties were analyzed. Confidence intervals (95%) of effect sizes were estimated for significance tests using the Z-statistic (α = 0.05 when a difference between groups is predicted and α = 0.20 when the equivalence of groups is predicted) (48). Positive values of within-effect sizes represent an observed improvement concerning the regarded dimension, with the exception of quality of life and body experience. The time points of post-intervention measurement and, consequently, the intervals between pre-intervention measuring and follow-up differed. Tests of heterogeneity were carried out using the Q-Statistic (relevant heterogeneity was assumed for *p* ≤ 0.20). As a quantitative measure, *I*^2^ indicates the percentage of variance that is attributable to study heterogeneity.

Subgroup analyses were carried out in case of significant heterogeneity for these dimensions: year of publication, sample size, diagnosis, bodily active control, and dose of therapy. Data management and statistical analyses were executed with the open-source online platform Covidence and the Review Manager 5.3 of the Cochrane Collaboration.

Concerning dropout, as a convention, sample size was fixed to the smallest available number of participants per dimension. For categorial data, odds ratios (and their SE) were calculated and transformed into SMD (and corresponding SE).

## Results

### Description of Studies

**Figure 1:**
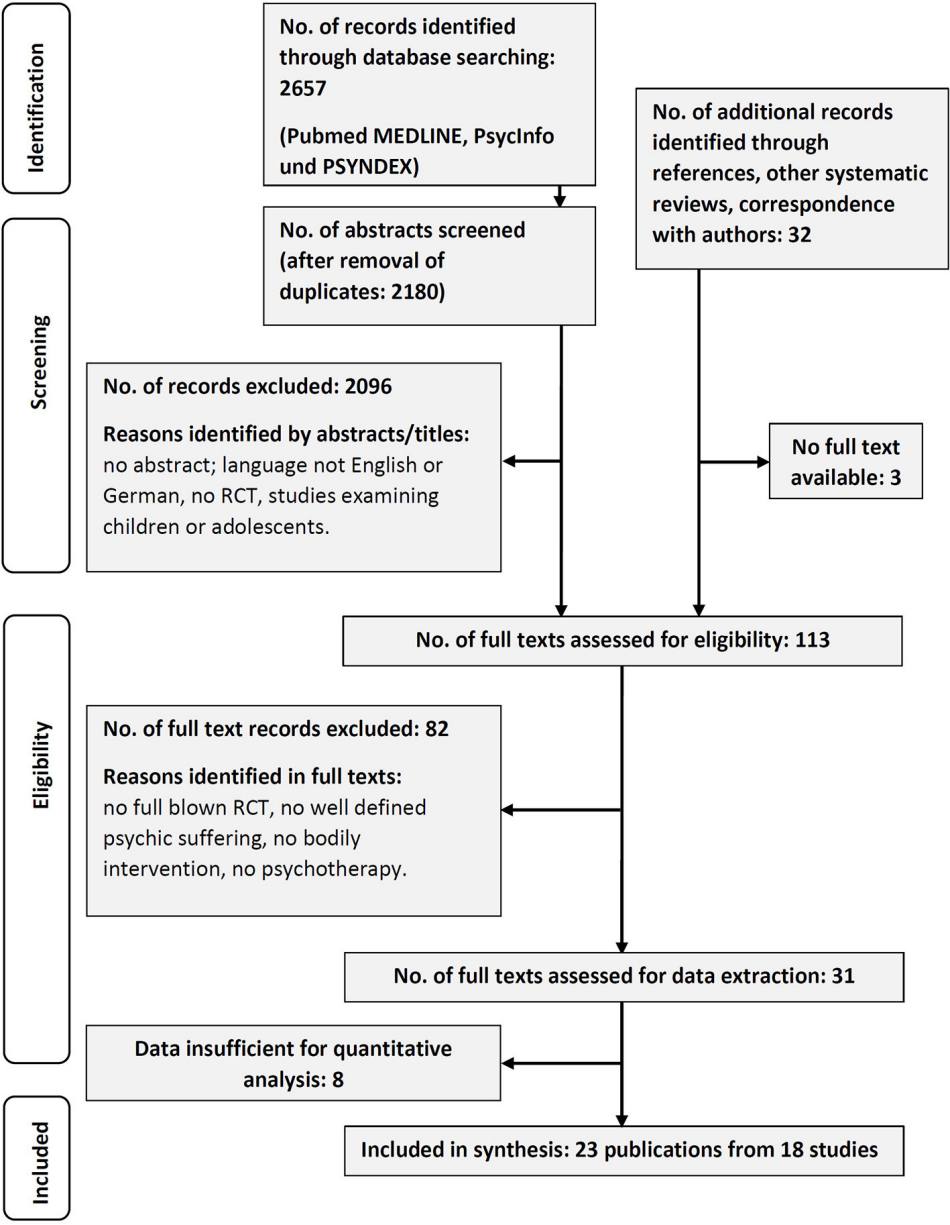
Flow of information through the phases of the review (Prisma 2009).

The literature search yielded 2,657 publications in three databases, whereof 2,180 remained after duplicates were removed. From these, 84 did meet the inclusion criteria. Another 32 records were identified by additional measures, whereof 3 records were not attainable in full text. One hundred thirteen publications were scrutinized in detail. Finally, 23 records from 18 studies were selected for meta-analysis (see Figure 1). Eight records could not be included, as they lacked important information for integration and analysis or offered physiological data only.

The characteristics of the included studies are summarized in Table 1. The whole sample consisted of 1,297 patients with a range of sample size over studies between 15 and 275 participants. Concerning type of intervention, three studies employed functional relaxation, one employed bio-energetics, five followed a manual on BPT (36), three followed a mindfulness-based protocol, two followed a common affect-focused root, three had background of psychomotor therapy, and one had an idiosyncratic approach (54). All but four studies applied BPT as a group therapy, often as a part of (multimodal) training programs (e.g., as adjunct therapy to psychotherapy and/or psychoeducation).

**Table 1:** Characteristics of included studies.

**Study**	**Title**	**Study population^a^**	**Intervention (format^b^, dose)**	**Control group activity (dose)**	**Outcomes (dimension: scale)**	**Quality score**
Boerhout et al. (51)	Effect of aggression regulation on eating disorder pathology: RCT of a brief body and movement-oriented intervention	*N* = 29; *N* (BPT) = 17; *N* (CG) = 12; m = 0; f = 29; Age M (SD) = 27.5 (8.4); eating disorder (anorexia nervosa, bulimia nervosa or eating disorder nos)	Body and movement -oriented intervention delivered by a psychomotor therapist (g (dyads), 1 h/week for 6 weeks)	Supportive contact weekly or biweekly including psychoeducation or nutrition counseling	Psychopathology: eating pathology (Eating Disorder Examination-Self-report Questionnaire, EDE-Q) Coping abilities: Anger In and Out (Self-expression and control scale, SECS)	26
Boerhout et al. (52)	Aggression regulation in day treatment of eating disorders: two-center RCT of a brief body and movement-oriented intervention	*N* = 70; *N* (BPT) = 38; *N* (CG) = 32; m = 3; f = 67; Age M (SD) = 24.2 (7.5); eating disorder (anorexia nervosa, bulimia nervosa or eating disorder nos)	Multidisciplinary day treatment plus body and movement-oriented intervention by a psychomotor therapist (g (dyads), 1 h/week; duration 6 weeks)	Multidisciplinary day treatment (3–5 days per week; duration 3–9 months)	Psychopathology: eating pathology (EDE-Q) Coping abilities: Anger In and Out (SECS)	36
Carletto et al. (53)	The effectiveness of a body-affective mindfulness intervention for multiple sclerosis patients with depressive symptoms: a randomized controlled clinical trial	*N* = 90; *N* (BPT) = 45; *N* (CG) = 45; m = 26; f = 64; Age M (SD) = 24.2 (7.5); depressive symptoms	Body-affective mindfulness intervention (g, one 3 h-session/week duration 8-weeks plus one additional 7 h-session and regular homework)	Psycho-educational intervention (one (one 3 h-session/week; duration 8-weeks)	Psychopathology: Beck depression inventory-II (BDI-II) + Beck anxiety inventory (BAI) Psychological distress: Fatigue Severity Scale (FSS) + Perceived Stress Scale (PSS) Quality of life: Functional Assessment of Multiple Sclerosis (FAMS)	35
Heimbeck and Hölter (54)	Movement therapy and depression—evaluation study of a disorder-orientated and an unspecific movement-therapeutic support in clinical context.	*N* = 98; *N* (BPT) = 53; *N* (CG) = 45; m = 41; f = 57; Age M (SD) = 47.7 (8.6); depression	Disorder-oriented movement therapy (g, 100 + 50 min meditation/week; duration on average 5 weeks)	Nordic walking (3 × /week for 50 min; on average for 5 weeks) + behavioral therapy (single and group session during inpatient treatment)	Psychopathology: Beck Depression Inventory (BDI)	25
Lahmann et al. (55)	Efficacy of functional relaxation and patient education in the treatment of somatoform heart disorders: A randomized, controlled clinical investigation	*N* = 22; *N* (BPT) = 11; *N* (CG) = 11; m = 10; f = 12; Age M (SD) = 44.35 (not reported); non-specific chest pain	Functional relaxation (g, 10 sessions for 90 min; duration 6 weeks)	Intensified medical care (medical standard treatment plus 2 × counseling)	Psychopathology: Symptom Checklist (SCL)- somatization + anxiety + Giessen Inventory of Complaints—cardiovascular complaints Psychological distress: SCL-GSI, Giessen Inventory of Complaints—total complaints Interpersonal difficulties: SCL-interpersonal sensitivity	29
Lahmann et al. (56)	Functional relaxation as complementary therapy in irritable bowel syndrome: A randomized, controlled clinical trial.	*N* = 80; *N* (BPT) = 40; *N* (CG) = 40; m = 27; f = 53; Age M (SD) = 48.8 (11.3); irritable bowel syndrome	Functional relaxation (g, 2 × /week for 60 min; duration 5 weeks)	Intensified medical care (medical standard treatment plus 2 × counseling)	Psychopathology: impairment-severity score (IS) Psychological distress: irritable bowel syndrome symptoms—subjective overall impairment; Interpersonal difficulties: IS—social impairment	33
Lahmann et al. (57)	A randomized controlled trial on functional relaxation as an adjunct to psychoeducation for stress	*N* = 81; *N* (BPT) = 42; *N* (CG) = 39; m = 18; f = 63; Age M (SD) = 47.2 (11.4); elevated stress levels	Functional relaxation and patient education (g, 90 min/week for 10 weeks)	Patient education (2 sessions of 90 min, second session 6 weeks after first one)	Psychopathology: Depression (PHQ-9) + somatization (PHQ-15) Psychological distress: Perceived Stress Questionnaire (PSQ)	30
Levy Berg et al. (58)	Affect-focused body psychotherapy in patients with generalized anxiety disorder: Evaluation of an integrative method.	*N* = 61; *N* (BPT) = 33; *N* (CG) = 28; m = 19; f = 42; Age M (Range) = 37.5 (21–58); generalized anxiety disorder	Affect-focused BPT (i, 1 × /week for 60 min, duration: 1 year)	TAU including no or low frequency contact with medical staff up to regular treatment including psychotherapy	Psychopathology: BAI + SCL-anxiety Psychological distress: SCL-GSI Quality of life: WHO Well-Being-Index (WWBI)	27
Martin et al. (59)	Overcoming disembodiment: The effect of movement therapy on negative symptoms in schizophrenia–A multicenter randomized controlled trial	*N* = 68; *N* (BPT) = 44; *N* (CG) = 24; m = 36; f = 32; Age M (SD) = 39.8 (10.4); schizophrenia	Movement therapy (BPT/Dance movement therapy) (g, 2 × /week for 90 min, duration 10 weeks)	Waiting condition, medication	Psychopathology: Scale of the Assessment of Negative Symptoms (SANS)-total score	26
Monsen and Monsen (60)	Chronic pain und psychodynamic body therapy: A controlled outcome study	*N* = 40; *N* (BPT) = 20; *N* (CG) = 20; m = not reported; f = not reported; Age M (Range) = 45 (29–61); somatoform pain disorder	psychodynamic BPT [i, on average 33 1-h sessions (range 15–41); duration = 9 months]	Waiting list	Psychopathology: Minnesota multiphasic personality inventory (MMPI) + pain-visual analog scale (VAS) Psychological distress: SCL-GSI Interpersonal difficulties: SCL-interpersonal sensitivity + inventory of interpersonal problems (IIP-C)	22
Nickel et al. (61)	Bioenergetic exercises in inpatient treatment of Turkish immigrants with chronic somatoform disorders: A randomized, controlled study	*N* = 128; *N* (BPT) = 64; *N* (CG) = 64; m = not reported; f = not reported; Age M (SD) = 48.9 (7.3); somatoform disorder	Inpatient treatment (including psychotherapy in first language, s and g) plus bioenergetic exercises (g, 2 × /week for 60 min; duration 6 weeks)	Inpatient treatment (same as intervention group) plus gymnastics in lieu of bioenergetic exercises	Psychopathology: SCL-somatization Psychological distress: SCL-GSI Coping abilities: State Trait Anger Expression Inventory (STAXI)-Anger Interpersonal difficulties: SCL-interpersonal sensitivity	27
Price (62)	Body-oriented therapy in recovery from child sexual abuse: An efficacy study	*N* = 24; *N* (BPT) = 12; *N* (CG) = 12; m = 0; f = 24; Age M (Range) = 41 (26–56); sexual abuse, engaged in psychotherapy	body-oriented therapy (i, 8 × for 60 min, weekly sessions) + ongoing psychotherapy	Massage (8 × for 60 min, weekly sessions) + ongoing psychotherapy	Psychopathology: Crime-related post-traumatic stress disorder scale + Dissociation experiences scale (DES) Psychological distress: Brief Symptom Inventory-GSI Body experience: Scale of body connection (SBC) and body investment scale (BIS)	26
Price et al. (63)	Mindful awareness in body-oriented therapy as an adjunct to women's substance use disorder treatment: A pilot feasibility study	*N* = 46; *N* (BPT) = 31; *N* (CG) = 15; m = 0; f = 46; Age M (Range) = 39 (19–58); substance use disorder	Elaborated treatment programme (TAU) plus mindful awareness in body-oriented therapy (i, 90 min/week for 8 weeks)	TAU: psycho-education and cognitive-behavioral therapy (3–5 week inpatient program, followed by a 12–24 week outpatient program 2–3 times/week for 3 h each)	Psychopathology: total abstinence Coping abilities: Dissociation, stress, affect, and emotion regulation difficulties Body experience: SBC + BIS	28
Priebe et al. (64)	Effectiveness of group body psychotherapy for negative symptoms of schizophrenia–A multicentre randomized controlled trial	*N* = 275; *N* (BPT) = 140; *N* (CG) = 135; m = 203; f = 72; Age M (SD) = 42.2 (10.7); schizophrenia	BPT following own manual (g, 2 × 90 min/week for 10 weeks)	Pilates (2 × 90 min/week; duration 10 weeks)	Psychopathology: Positive and negative syndrome scale (PANSS)-negative scale, PANSS Marder (factor solution)-negative scale, Clinical assessment interview for negatives symptoms (CAINS) Psychological distress: PANSS general + Calgary depression scale Quality of life: Manchester short Assessment of Quality of life (MANSA)	40
(a) Röhricht et al. (65)(b) Papadopoulos and Röhricht (66)	(a) An exploratory randomized controlled trial of body psychotherapy for patients with chronic depression (b) An investigation into the application and processes of manualised group body psychotherapy for depressive disorder in a clinical trial	*N* = 31; *N* (BPT) = 16; *N* (CG) = 15; m = 18; f = 13; Age M (SD) = 47.7 (10.4); depression	BPT following own manual (g, 2 × /week for 90 min; for 10 weeks)	Waiting condition	Psychopathology: Hamilton rating scale (HAMD) Quality of life: MANSA Body experience: Body image und Body cathexis/satisfaction	29
Röhricht and Priebe (67)	Effect of body-oriented psychological therapy on negative symptoms in schizophrenia: A randomized controlled trial	*N* = 45; *N* (BPT) = 24; *N* (CG) = 21; m = 22; f = 23; Age M (SD) = 38.3 (9.4); schizophrenia	BPT following own manual (g, 2 × /week for 60–90 min; for 10 weeks)	Supportive counseling	Psychopathology: PANSS-negative scale Psychological distress: PANSS general Quality of life: MANSA	37
Röhricht et al. (68)	Group body psychotherapy for the treatment of somatoform disorder—a partly randomized-controlled feasibility pilot study	*N* = 15; *N* (BPT) = 7^c^; *N* (CG) = 8; m = 4; f = 11; Age M (SD) = 50 (11.1); somatoform disorder	BPT following own manual (g, 20 weekly group sessions for 90 min, duration 4 to 6 months)	Waiting condition	Psychopathology: PHQ-somatic symptom score + Somatic symptom screening scale (SOMS)-number of symptoms Psychological distress: PHQ-depression, somatization, stress + SOMS Quality of life: SF-36 physical and mental Body experience: Dresden-Body-Image questionnaire (DKB-35) + Body Distortion Questionnaire (BDQ)	24
van der Maas et al. (69)	Improving the multidisciplinary treatment of chronic pain by stimulating body awareness A cluster-randomized trial	*N* = 94; *N* (BPT) = 49; *N* (CG) = 45; m = 17; f = 77; Age M (SD) = 41.9 (11.1); chronic musculoskeletal pain	Multicomponent training plus additional psychomotor therapy (g, 10 × 1.5 h)	Multicomponent training (total of 94 h; duration 12 weeks)	Psychopathology: BDI Psychological distress: Pain Disability Index (PDI) Quality of life: SF-36 physical + mental component score Coping abilities: Pain catastrophizing scale	29

a *The listed sample sizes correspond to the respective sizes stated in the original articles when demographic data is presented*.

b *Format of intervention is coded as g = group and i = individual intervention*.

c *Fifteen patients were randomly assigned to receive either manualized BPT or TAU, (seven patients directly assigned to BPT not included in the present analysis)*.

*BAI, Beck anxiety inventory; BDI-II, Beck depression inventory-II; BIS, body investment scale; BDQ, Body Distortion Questionnaire; CAINS, Clinical assessment interview for negatives symptoms; DES, Dissociation experiences scale; DKB-35, Dresden-Body-Image questionnaire; EDE-Q, Eating Disorder Examination-Self-report Questionnaire; FAMS, Functional Assessment of Multiple Sclerosis; FSS, Fatigue Severity Scale; GSI, Global severity index; HAMD, Hamilton rating scale; IIP-C, Inventory of Interpersonal Problems; IS, Impairment severity score; MANSA, Manchester short Assessment of Quality of life; MMPI, Minnesota multiphasic personality inventory; PANSS, Positive and negative syndrome scale; PDI, Pain Disability Index; PHQ, Patient Health Questionnaire; PSQ, Perceived Stress Questionnaire; PSS, Perceived Stress Scale; SBC, Scale of body connection; SANS, Scale of the Assessment of Negative Symptoms; SCL-90, Symptom-Checklist 90; SCL-GSI, global severity index of the symptom checklist; SECS, self-expression and control scale; SF-36, short form 36; SOMS, Somatic symptom screening scale; STAXI, State Trait Anger Expression Inventory; WWBI, WHO Well-Being-Index*.

### Effects of Interventions

**Figure 2:**
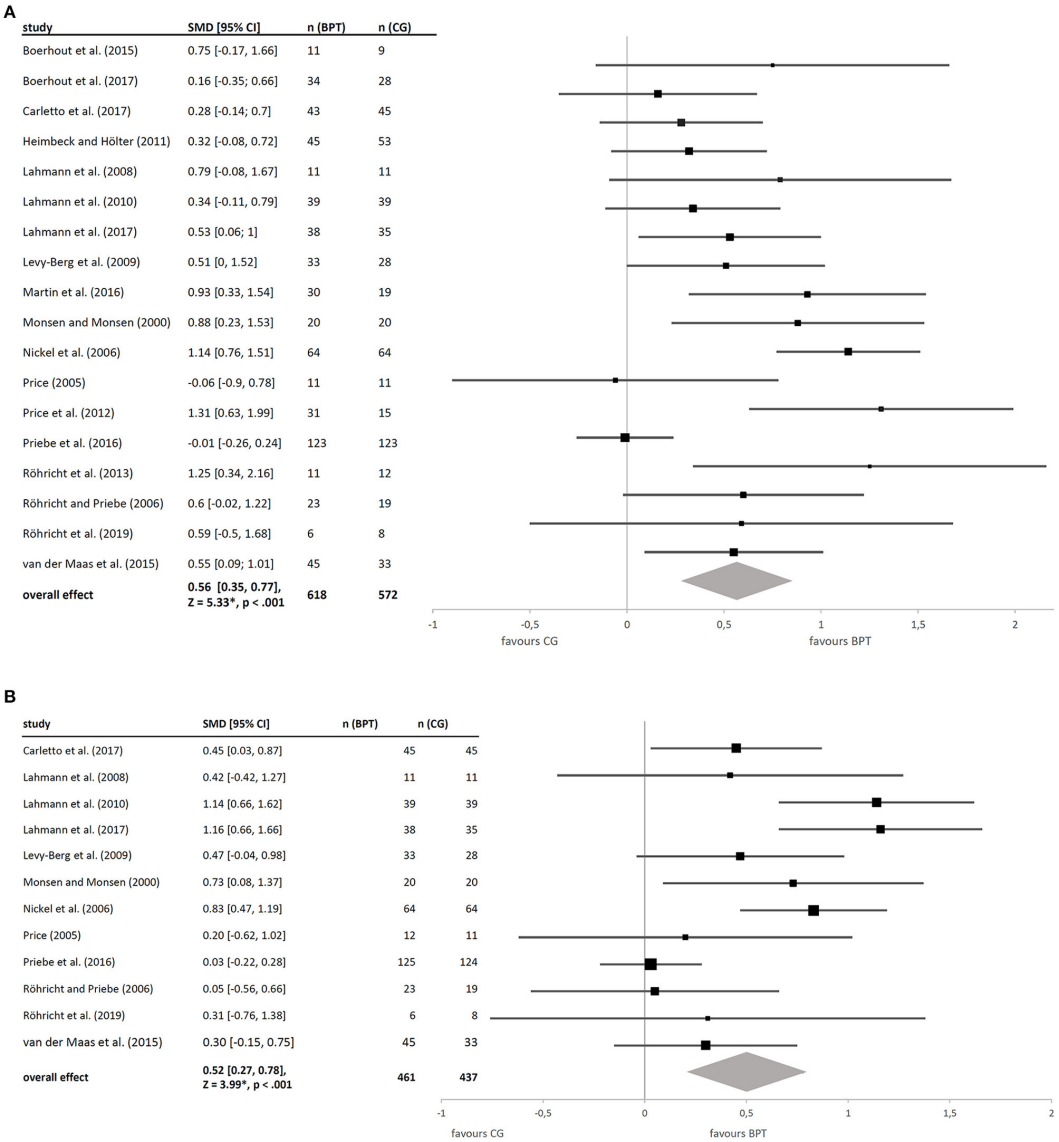
**(A)** Forest plot of between-group effect (BPT vs. CG) on psychopathology. **(B)** Forest plot of between-group effect (BPT vs. CG) on psychological distress.

Changes in psychopathology as the most frequently examined outcome dimension were reported for 18 studies (Figure 2A). The majority of those studies (16, 88.9%) suggests that BPT—in comparison to the respective control condition—brings about an improvement in psychopathology, which was statistically significant in eight studies. No improvement at all was observed in two studies applying an equally dosed physical activity for the control group (54, 62). The total effect size is medium {*g* = 0.56 [95% CI (0.35, 0.77)], *Z* = 5.33, *p* < 0.001} with a moderate to high degree of heterogeneity [τ^2^ = 0.11, ${\text{χ}}^{2_{\left(17\right)}}$ = 44.97, *p* < 0.001, *I*^2^ = 62%].

For psychological distress (Figure 2B), included studies demonstrated an—at least—small advantage of BPT as compared to control groups, which results in a moderate total effect [*g* = 0.52, 95% CI (0.27, 0.78), *Z* = 3.99, *p* < 0.001], again with moderate to high heterogeneity [τ^2^ = 0.13, ${\text{χ}}_{\left(11\right)}^{2}$ = 34.06, *p* < 0.001, *I*^2^ = 68%]. Moreover, the corresponding within-group effects on the primary outcomes are statistically significant (for details, contact authors).

### Secondary Outcomes

**Figure 3:**
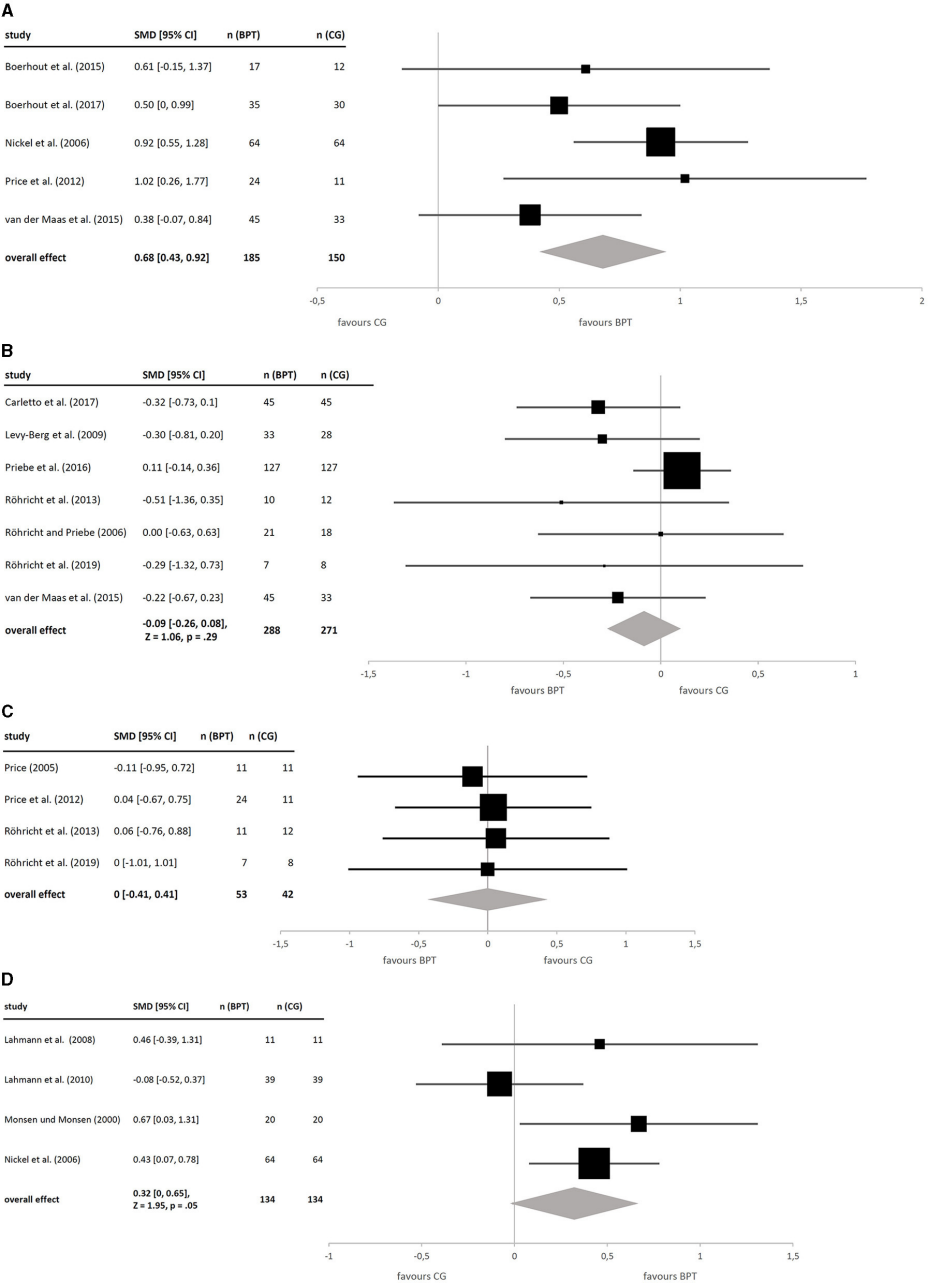
**(A)** Forest plot of between-group effects (BPT vs. CG) on coping abilities. **(B)** Forest plot of between-group effects (BPT vs. CG) on quality of life. **(C)** Forest plot of between-group effects (BPT vs. CG) on body experience. **(D)** Forest plot of between-group effects (BPT vs. CG) on interpersonal conflicts.

Data on coping abilities, quality of life, body experience, and interpersonal difficulties were available for a restricted number of studies only (see Figures 3A–D). On coping, five studies showed a moderate to high homogeneous total effect size for BPT, which was statistically significant [*g* = 0.68, 95% CI (0.43, 0.92), *Z* = 5.42, *p* < 0.001]. As the effects on primary outcomes, this effect relies on a more pronounced amelioration of the intervention groups. At the same time, an effect of BPT on quality of life could not be demonstrated [*g* = −0.09, 95% CI (−0.26, 0.08), *Z* = 1.06, *p* = 0.29, n. s.] in seven included studies, although five of these studies revealed an at least slight improvement (represented by a numerically negative effect) in this dimension. In four studies reporting data for body experience, an improvement for BPT could not be demonstrated [g = 0, 95% CI (−0.41, 0.41), *Z* = 0, *p* = 1.00, n. s.]. As to interpersonal conflicts, the four analyzed studies suggested a small effect size, indicating a benefit of BPT compared to CG bordering statistical significance [*g* = 0.32, 95% CI (0, 0.65), *Z* = 1.95, *p* = 0.05, n. s.].

### Subgroup Analyses Investigating Heterogeneity

**Figure 4:**
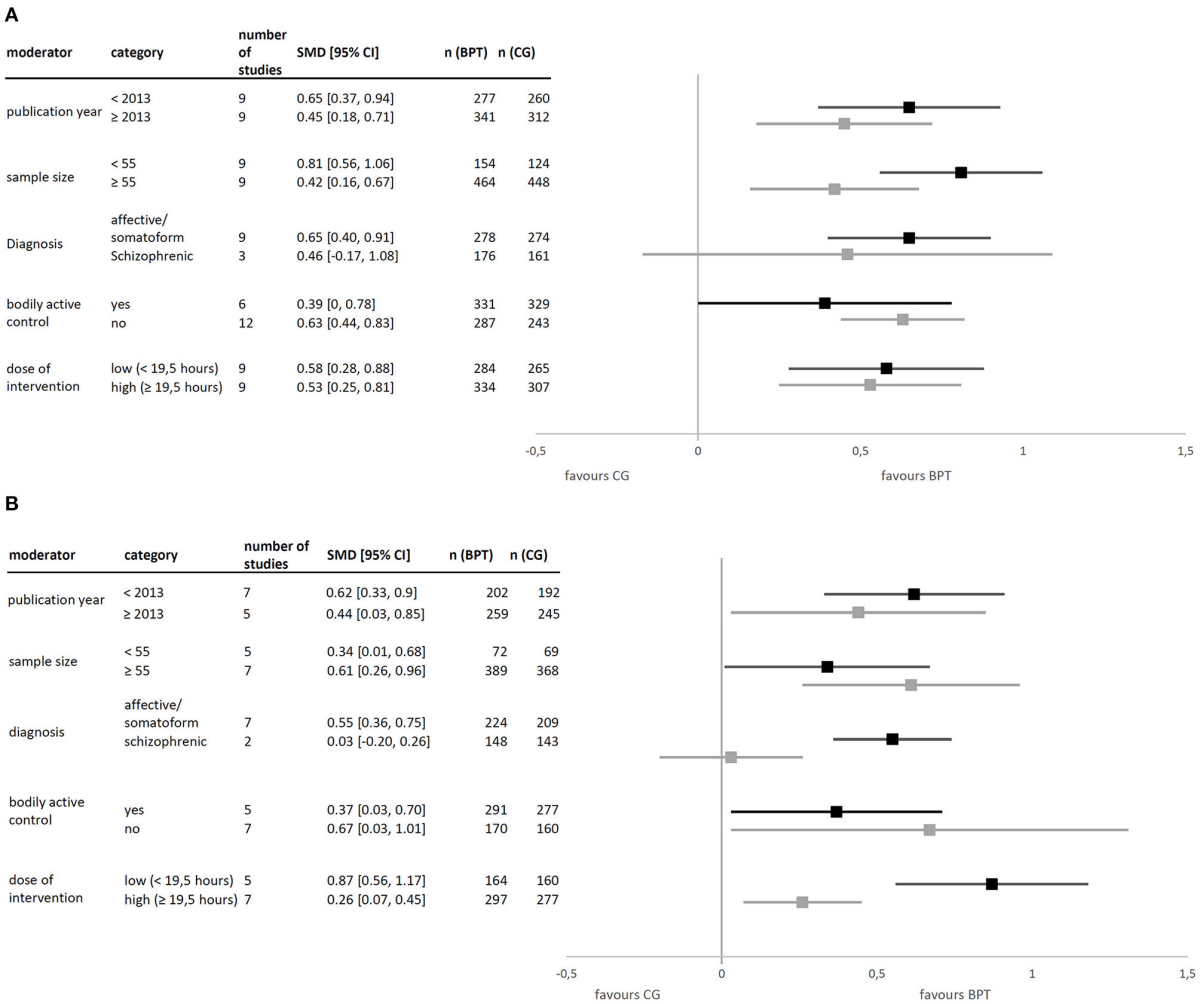
**(A)** Effect on psychopathology split on different moderators. **(B)** Effect on psychological distress split on different moderators.

*Post-hoc*, we performed subgroup analyses on within-group effects in primary outcomes (see Figures 4A,B). Categorial moderators divided studies naturally into two groups, whereas median splits were applied for continuous moderators based on all studies. Due to the exploratory character of these analyses, we did not interpret tests of significance. It should be noted that we did not investigate interdependence of moderators.

Studies published before 2013 had a slightly higher effect than those published later on both primary outcomes. Concerning sample size, results were inconsistent across primary outcomes. With regard to diagnosis, 12 studies allowed a distinction between effects for patients with affective and somatoform disorders as compared to those with schizophrenia, with smaller and more heterogenous effects for the latter. In studies with an active control group, the advantage of BPT diminishes, as compared with inactive CGs (e.g., waiting list). As to dose, a smaller number of intervention hours corresponded to a larger effect.

### Catamnestic Data

To estimate long-term stability of the effects, we integrated catamnestic data. On average, follow-up was conducted 7 months (range, 3–13) post-intervention. For psychopathology, the long-term total effect-size slightly decreased to a small to medium effect [*g* = 0.41, 95% CI (0.14; 0.67), *Z* = 2.99, *p* < 0.01] with 10 included studies [*n* (BPT) = 384, *n* (CG) = 369]. However, for psychological distress, the effect remained quite stable with *g* = 0.47 [CI 95% (0.22; 0.72), *Z* = 3.75, *p* < 0.001] induced from eight studies [*n* (BPT) = 319, *n* (CG) = 306], with both effects statistically significant.

### Evaluation of Publication Bias

In order to account for possible publication bias, we generated funnel plots on primary outcomes (see Supplementary Figures 1A,B). As for psychopathology, studies gather symmetrically around the estimated common effect. However, as the supposed triangle is missing its top, there is a lack of studies with bigger sample sizes. The distribution of studies for psychological distress shows an asymmetrical form, suggesting a loss of smaller studies with more pronounced effects.

## Discussion

This systematic review and meta-analysis aimed to give an overview of existing randomized controlled trials on BPT. In summary, we found moderate effect sizes for BPT on the selected primary outcomes psychopathology and psychological distress, which are characterized by substantial statistical heterogeneity of the included studies. This may reflect the wide variety of the examined interventions, study designs, and included diagnoses. However, for the dimensions investigated, nearly all studies reported effects in favor for BPT. Exploratory subgroup analyses suggest potential influences especially of study design and diagnosis.

### Effect on Primary Outcomes

The effect size on primary outcomes corresponds to an NNT of 4 (24) that is comparable to the one found by Koch et al. (42) for dance and movement therapy (*g* = 0.34–0.46) on symptomatology. In comparison, for mindfulness-based interventions (41), an effect of 0.73 was found on depressive symptoms. All effects integrated in our study remain smaller than the commonly assumed effect of psychotherapy (0.80) when compared to a control without any treatment. A possible explanation with regard to heterogeneity is that, indeed, different effects, respectively, treatments and comparisons, are summarized. This counts as well for study designs—additive designs investigating BPT as a part of an already established multimodal treatment will not be likely to reveal large effect sizes. Heterogeneity affects within-group CG effects as well. Accordingly, the extensive variability of the chosen CGs is underlined by the corresponding subgroup analyses.

### Subgroup Analyses

Subgroup analyses could be performed for common factors reported by all or at least most included studies. Study size influences both primary outcomes differently, as the effect decreases with increasing study size only on psychopathology, not on psychological distress. This might be seen as an argument against publication bias.

Concerning diagnosis, the diminished effect in schizophrenia seems to be common for different types of psychotherapy (70–72), which reflects, on the one hand, the general difficulty to treat this disorder. On the other hand, it underlines the need to develop diagnosis-specific therapeutic techniques, as an effect of BPT on positive symptoms could not have been shown so far (64, 67). The result that, compared to men, women might benefit from BPT with regard to body expressiveness (73), hints to the need for individualized therapies.

As to study design, comparisons with CGs that followed a physical activity (e.g., Pilates, massage) led to smaller between-group effects of BPT on primary outcomes than comparisons with inactive controls. Because of the small number of studies, we did not differentiate between exercise only and body therapy (techniques that focus on the experienced body to increase the person's physical and psychological well-being) as control conditions. A medium effect of exercise could be found on depressive symptoms (74); yoga might even have a small advantage compared to exercise and be—combined with pharmacotherapy—as helpful as psychotherapy (75). Interestingly, the absolute size of within-group effects varies strongly between primary outcomes when applying this moderator, which calls for further studies to clarify its role. A dependence of effect on activity of control group was found for mindfulness-based interventions (41) and poses a known threat to the proof of specific effectiveness of psychotherapy (76, 77). The ongoing debate about the impossibility of constructing an inert placebo stresses the importance of establishing common work factors (78) as might be embodiment. Further on, the interpersonal work on body experience, if fitted to specific disorders, could be an additive that qualifies a psychotherapy as opposed to body therapies without this component.

Surprisingly, BPT interventions with smaller dose (hours of therapy) produced larger effects than those with higher dose, an observation that cannot be confirmed by other meta-analyses (76, 79). As a confounder, the difference in periods between pre- and post-treatment measurements has to be taken into account. While low-dose interventions typically lasted 5–12 weeks, high-dose interventions took 8–60 weeks. Consequently, post-treatment measuring of the former might benefit from the natural course of mental disorders. For example, an untreated depressive episode usually lasts ~2–3 months (80), so that remission might fall together with post-treatment measuring of low-dose interventions. On the other hand, the diminished effect of high-dose and longer lasting interventions could mirror the delayed occurrence of an aversive reaction when applying BPT that might be a necessary step in a healing process (81).

### Secondary Outcomes

Secondary outcomes were tested to analyze potential mediators of BPT. The large, homogeneous effect on coping abilities should not be granted as a general effect because of the small number of studies that it relies on. Nevertheless, this finding might be seen as a positive indicator that BPT targets emotion regulation. While other meta-analytic data on coping is rare, an evaluation on psychosocial group interventions that addressed coping reported no amelioration (82). Consequently, there is a need of integrating process variables in meta-analytic research, including a differentiation of positive and negative coping styles (83, 84).

Considering quality of life, both groups benefited equally, which contradicts meta-analyses on other types of psychotherapy (85). When comparing different diagnoses, it is compelling that in the present review, studies concerning depression and anxiety disorders typically report a more pronounced amelioration than those with schizophrenia.

Neither did we find a difference concerning body experience, where comparable evidence is rare as well. In this regard, the distinction between body schema and body image by Gallagher (14) might be helpful. Thus, on a theoretical level, it might not be adequate to combine scales that measure each just one aspect, as they may follow different rationales and/or time courses, for example. However, for interpersonal problems, we found a slight advantage of BPT that bordered significance and was comparable to the effects of other psychotherapies (42, 86).

### Shortcomings

General concerns regarding meta-analysis potentially limit the usefulness of the applied meta-analytic approach when informing patient care (87). In particular, a risk of bias in this meta-analysis remains. Indeed, the respective funnel plots did not indicate absence of any bias for each outcome. These findings underline the need for high-quality studies with bigger sample sizes to estimate the effect more precisely. As to study identification, the search term might be criticized because it favors studies of a certain therapy tradition by employing typical vocabulary. Consequently, it cannot be ruled out that types of therapy that are similar in content but use a different wording are neglected. In addition, plausible moderators should be entered in a multiple regression to address possible interactions. While the overall number should be reduced, it might be interesting to differentiate between dose and length and consider the severity of disorder. Finally, the scrutinized evidence was too small to statistically test potential process variables and moderators.

### Practical Consequences

The evidence identified in this study suggests that BPT often was applied as adjunct therapy rather than an alternative to single psychotherapy. Accordingly, BPT often was examined in (multimodal) treatment programs for following an additive research design. This issue might result in some benefit for external validity. Moreover, the most commonly used group setting may improve efficacy and economical implementation of these approaches.

## Conclusions

This study scrutinizes psychotherapeutic approaches providing psychological and bodily interventions simultaneously, studies that accordingly could be characterized as implementing an “embodiment” perspective. The analysis could demonstrate at least some non-neglectable evidence for the effectiveness of BPT: the meta-analytic integration of the observed effect sizes revealed moderate effects of BPT on the chosen primary outcomes psychopathology and psychological distress, indicating that the examined studies provided potentially efficacious measures.

For a better understanding of the mechanisms under question, we carried out subsequent subgroup analyses. Most evidently, the good performance of, in particular, active control groups should not be underestimated. As a consequence, BPT and related approaches appear as good candidates to further develop disorder-specific treatment approaches. However, the potential occurrence of negative side effects of BPT, for example, on trauma, should not be neglected further. Reflecting the rich variety of applied therapeutic approaches in very heterogeneous domains of psychic suffering and variegated treatment settings, there seems to be a strong need for future, high-quality studies with bigger samples to elucidate conditions for the effective use of BPT. We emphasize that those studies should report data on safety aspects, adverse events, acceptability, and patients' preferences, too.

## Data Availability Statement

The original contributions presented in the study are included in the article/Supplementary Material, further inquiries can be directed to the corresponding author/s.

## Author Contributions

All authors listed have made a substantial, direct and intellectual contribution to the work, and approved it for publication.

## Conflict of Interest

The authors declare that the research was conducted in the absence of any commercial or financial relationships that could be construed as a potential conflict of interest.

## Publisher's Note

All claims expressed in this article are solely those of the authors and do not necessarily represent those of their affiliated organizations, or those of the publisher, the editors and the reviewers. Any product that may be evaluated in this article, or claim that may be made by its manufacturer, is not guaranteed or endorsed by the publisher.

## Supplementary Material

Supplementary Figure 1 **(A)** Funnel plot on psychopathology. **(B)** Funnel plot on psychological distress.

Supplementary Table 1 Assessment of Study Quality.
