# Supplementary material for: Effectiveness of Body Psychotherapy. A Systematic Review and Meta-Analysis
Source: Front Psychiatry. 2021 Sep 9;12:709798. doi: 10.3389/fpsyt.2021.709798 (PMC8458738; doi:10.3389/fpsyt.2021.709798)
Supplement: Supplementary Figure 1 — (A) Funnel plot on psychopathology. (B) Funnel plot on psychological distress. [file Image_1.pdf]

(A) Psychopathology

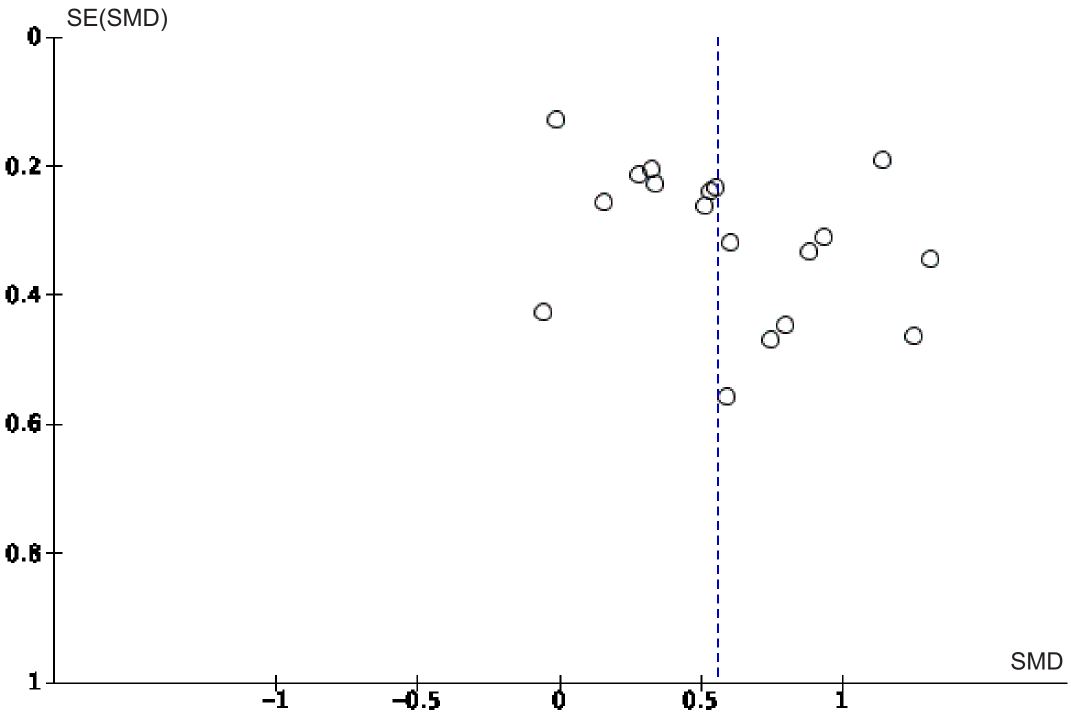

(B) Psychological Distress

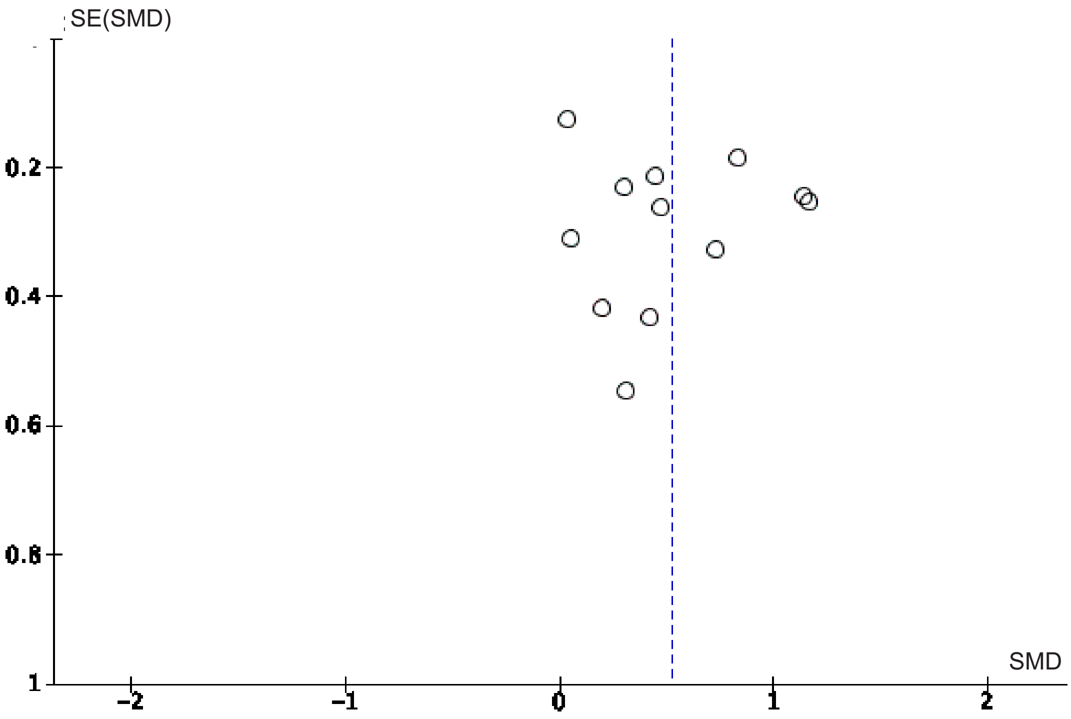

Supplementary Figure 1 | (A) Funnel plot on psychopathology. (B) Funnel plot on psychological distress.
