# Supplementary material for: Effectiveness of Body Psychotherapy. A Systematic Review and Meta-Analysis
Source: Front Psychiatry. 2021 Sep 9;12:709798. doi: 10.3389/fpsyt.2021.709798 (PMC8458738; doi:10.3389/fpsyt.2021.709798)
Supplement: Supplementary Table 1 — Assessment of Study Quality. [file Table_1.pdf]

Study	Boerhout et al. (2015)	Boerhout et al. (2017)	Carletto et al. (2017)	Heimbeck and Hölter (2011)	Lahmann et al. (2008)	Lahmann et al. (2010)	Lahmann et al. (2017)	Levy-Berg et al. (2009)	Martin et al. (2016)	Monsen And Monsen (2000)	Nickel et al. (2006)	Price (2005)	Price et al. (2012)	Priebe et al. (2016)	Röhricht et al. (2013)	Röhricht and Priebe (2006)	Röhricht et al. (2019)	van der Maas et al. (2015)
1) Objectives and specification main outcomes a priori	2	2	2	2	2	2	1	2	2	1	2	2	2	2	2	2	2	1
2) Adequate sample size (n per group)	0	2	2	0	0	0	2	0	0	0	1	0	0	2	0	0	0	1
3)Appropriate duration of trial including follow up	0	0	2	2	0	1	2	2	0	2	0	2	2	2	0	1	0	2
4) Power calculation	2	2	2	0	2	2	1	0	2	0	2	0	0	2	0	2	0	0
5) Method of allocation	2	2	2	1	2	2	2	2	2	1	2	2	2	2	2	2	2	2
6) Concealment of allocation	2	2	2	0	2	2	2	0	2	0	0	0	0	2	2	2	0	2
7) Clear description of treatments	1	2	2	1	2	2	2	1	2	1	1	2	2	2	2	2	2	1
8) Blinding of subjects	0	0	0	0	0	0	0	0	0	0	0	0	0	0	0	0	0	0
9) Source of subjects described and representative sample recruitment	2	2	2	2	2	2	1	2	2	1	2	1	1	2	2	2	2	2
10) Use of diagnostic criteria	1	2	2	2	1	1	2	1	1	1	1	1	1	2	2	2	1	1
11) Record of exclusion criteria and number of exclusions und refusals	2	2	2	1	1	1	1	1	2	2	2	1	2	2	2	2	1	2
12) Description of sample demographics	1	2	2	1	2	2	1	2	2	2	2	2	2	2	1	2	2	2
13) Blinding of assessor	0	0	0	0	0	1	0	0	1	2	0	0	0	2	0	1	0	0
14) Assessment of compliance with experimental treatments (including	0	2	0	0	0	0	0	0	0	0	0	0	2	2	2	2	2	0
15) Details on side-effects	0	0	0	0	0	0	0	0	0	0	0	0	0	1	0	1	0	0
16) Record of number and reasons for withdrawal by group	2	2	2	0	2	1	1	2	2	0	1	1	1	2	1	2	1	2
17) Outcome measures described clearly	2	2	2	2	2	2	2	2	2	2	2	2	2	2	2	2	2	2
18) Information on comparability and adjustment for differences in	2	2	2	2	2	2	2	2	1	2	2	2	2	1	2	1	2	2
19) Inclusion of all subjects in analyses, intention to treat	0	2	0	2	2	2	0	2	0	0	2	2	0	0	0	2	0	0
20) Presentation of results with inclusion of data for reanalysis for	2	2	2	2	2	2	2	2	2	2	2	2	1	2	2	2	2	1
21) Appropriate statistical analysis (correction for multiple testing)	1	2	1	1	1	2	2	2	0	1	2	2	2	2	1	1	1	2
22) Conclusions justified	2	2	2	2	2	2	2	2	1	2	1	2	2	2	2	2	2	2
23) Declaration of interests	0	0	2	2	0	2	2	0	0	0	0	0	2	2	2	2	0	2
**Sum score**	**26**	**36**	**35**	**25**	**29**	**33**	**30**	**27**	**26**	**22**	**27**	**26**	**28**	**40**	**29**	**37**	**24**	**29**

Supplementary Table1 | Assessment of Study Quality
